# Promoting Positive Affect through Smartphone Photography

**DOI:** 10.1186/s13612-016-0044-4

**Published:** 2016-07-04

**Authors:** Yu Chen, Gloria Mark, Sanna Ali

**Affiliations:** Department of Informatics, University of California, Irvine, 92697 USA

**Keywords:** Happiness, Photos, Smartphones, Positive affect, In situ study, Positive computing, Mental health

## Abstract

**Background:**

With the increasing quality of smartphone cameras, taking photos has become ubiquitous. This paper investigates how smartphone photography can be leveraged to help individuals increase their positive affect.

**Methods:**

Applying findings from positive psychology, we designed and conducted a 4-week study with 41 participants. Participants were instructed to take one photo every day in one of the following three conditions: a selfie photo with a smiling expression, a photo of something that would make oneself happy and a photo of something that would make another person happy.

**Findings:**

After 3 weeks, participants’ positive affect in all conditions increased. Those who took photos to make others happy became much less aroused. Qualitative results showed that those in the selfie group observed changes in their smile over time; the group taking photos to improve their own affect became more reflective and those taking photos for others found that connecting with family members and friends helped to relieve stress.

**Conclusions:**

The findings can offer insights for designers to create systems that enhance emotional well-being.

## Background

Consistently living under stress can lead to chronic health problems such as depression, anxiety disorders, heart disease, high blood pressure and diabetes (NIMH 2015). College students in particular are a vulnerable population that experience stress. Their stress may come from living away from family for the first time, feeling lonely or isolated, experiencing pressure from coursework, or worrying about finances (Mark et al. 2014; NIMH 2015). Stress is reported as one of the factors that negatively impacts students’ academic performance and thus can lead to depression (ACHA 2014). Conventional methods to cope with stress include medication, exercise, therapy and seeking emotional support (NIMH 2015).

Psychologists have investigated various methods of improving emotional and mental well-being. For example, writing down three things that went well during the day can significantly help people increase their level of happiness (Seligman et al. 2005). Dunn et al. (2008) found that people were happier when they spent money on others instead of on themselves. Embodying happiness—representing happiness in a physical form—can even relieve stress: a study showed that people became less stressed if they adopted a smiling facial expression (Kraft and Pressman 2012).

The last decade has witnessed the emergence of positive computing—the use of information technology to support human well-being (Calvo and Peters 2014). Researchers from diverse fields, such as psychology, social science, psychiatry and information science are bringing their expertise to leverage the ever-increasing advancement of informatics to help people better manage their emotional well-being (Seligman et al. 2005; Calvo and Peters 2014). At the same time, the high adoption of smartphones and social media brings new opportunities for measuring and sharing emotions. With the increasing quality of smartphone cameras, taking photos has become ubiquitous. This trend is reflected by the widespread popularity of photo-related social media such as Instagram, Snapchat and Facebook.

In this study, we investigated how we could leverage findings from positive psychology to promote people’s positive affect and potentially reduce stress through taking photos. We compared the impacts of photo-taking on well-being in three different conditions: (1) self-perception, in which people manipulated positive facial expressions; (2) self-efficacy, in which people did things to make themselves happy; and (3) pro-social, in which people did things to make other people happy. We developed two Android applications as experimental platforms that prompted users to take photos and report their mood. We then conducted a 4-week in-situ study to assess the effectiveness of taking photos to promote positive affect.

This work contributes to the field of positive computing in the following ways. First, it empirically demonstrates the effectiveness of using smartphone photography to promote positive affect. Second, it applies strategies that have been used in positive computing studies. Third, it offers implications for the design of systems that use smartphone photography to promote users’ emotional well-being.

## Related Work

Emotional well-being is an essential part of mental health (Ryff and Keyes 1995). Positive emotions are found to enhance cardiovascular, hormonal and immune functions, promote healthy behaviors such as better sleep and more exercise (Kraft and Pressman 2012), and lead to more open-minded thinking and effective problem solving (Calvo and Peters 2014). In line with the importance of positive emotions, positive psychology emerged as a discipline with psychologists seeking to find various methods to help people increase their emotional well-being (Biswas-Diener and Dean 2010; Fredrickson 2001; Seligman et al. 2005; Sin and Lyubomirsky 2009). Meanwhile, the advent of pervasive sensors, wearable devices and mobile technologies has given rise to positive computing, the use of informatics to support mental well-being (Calvo and Peters 2014). In the area of affective computing (Picard 1997), researchers have employed various sensors to detect users’ affective states from facial expressions, speech, body gestures, or breath and then have presented visualizations of these states to users (Fernandez and Picard 2005; Kisacanin et al. 2005; Bernhardt and Robinson 2007; Spire 2015). Such methods can increase users’ awareness of their emotions and can trigger users to self-regulate, especially if they are experiencing negative affective states. However, the above monitoring methods lack an emphasis on empowering users to proactively change their emotions.

In this work, we set out to use technology to help users complete exercises designed to increase their positive affect. We chose smartphone photography as a means to make such practices accessible and habitual in people’s daily lives. Smartphone photography has been used as a memory aid, such as taking snapshots of price tags, recipes and maps (Häkkilä et al. 2012), as a tool to document life events (Lehtimäki 2008), and as media to communicate with friends and families (Cui et al. 2013). Sharing photos on social media has become widespread (Miller and Edwards 2007), as evidenced in the rise of Instagram, Snapchat and photo-sharing services on Facebook and Twitter (Hu et al. 2014). As of 2016, Instagram has over 400 million monthly active users and 80 million photos are uploaded every day (Instagram 2015). Among the photos uploaded, selfies—self-portraits made with a smartphone (Saltz 2014)—are becoming a wide-spread phenomenon. We sought to investigate and leverage findings of positive psychology to promote positive affect into the practice of taking photos. Particularly, we apply the following three theories that have been shown to improve people’s positive affect.

### Smiling Brings Happiness

Self-perception theory states that how people behave will determine what they think and how they feel (Bem 1973). In one study users became mentally stronger when they embodied body postures that were physically expansive and implied power (Cuddy 2012). In another study, participants who maintained a positive facial expression while stressed experienced less decrease in positive affect than those in a baseline group (Kraft and Pressman 2012). This study also demonstrated lower heart rates during stress recovery and the enhanced ability to endure stressful events. Kleinke et al. (1998) found that participants who engaged in positive facial expressions increased their positive mood. The effects were greater when participants viewed themselves in a mirror. Based on this theory, Tsujita and Rekimoto (2011) designed HappinessCounter, a device that recognizes users’ smiles, counts the number of smiles and then provides feedback in a mirror. Their field study showed that users became happier and smiled more naturally after ten days. The Mood Meter (Hernandez et al. 2012), which also encourages smiling of passersby in public places, consists of a camera that captures people’s facial expressions, a computer that analyzes the facial expressions and detects smiles, and a public display that shows users’ smiles. SmileTracker (Jaques et al. 2015) not only detects users’ smiles from the web camera of their computers, but also captures a screenshot of their smile for them to reflect upon that image in the future.

### Reflecting Brings Happiness

The “three-good-things-in-life” exercise, proposed by Seligman et al. (2005), asks participants to write down three things that went well that day and their causes. Participants became happier and less depressed after a one-month intervention. By implementing this exercise in online social networks, Munson et al. (2010) developed a Facebook application called HappierTogether. Another application inspired by this finding is Happier, a commercial mobile app that aims to promote users’ positive affect by having them take pictures to savor moments of happiness, reflect on the reason for such happiness and then keep the photo privately or share it with their social networks (Happier 2015). Similarly, Happify, another commercial website, also encourages users to record good things that happened each day using gamification (Happify 2015).

### Giving Brings Happiness

An experiment conducted by Dunn et al. (2008) showed that participants in an experimental condition where they spent money on others reported a higher degree of happiness than participants who were given instructions to spend money on themselves. In a study of Seligman et al. (2005), participants were instructed to write and then deliver a letter of gratitude in person to someone who had been kind to them but had never been properly thanked. The participants significantly increased their sense of happiness and this effect remained after 6 months. Mortality was even shown to be reduced for older adults who had reported providing instrumental and emotional support to their strong ties (Brown et al. 2003). The above findings suggest that giving to others can bring benefits to health and longevity.

In this section, we surveyed sensing technologies for monitoring emotional states, practices that promote positive well-being and technological tools that are created based on these practices. However, the practicality of the above tools (e.g., whether they can be adopted) has not been addressed. Our interest was in investigating how practical tools that have already been adopted could be used to enhance happiness. We thus chose to use smartphone photography as a medium to implement findings from research in the area of positive psychology. To our knowledge, this paper presents the first study that explores and compares the application of theories to promote happiness using smartphone photography.

## Methods

We conducted a 4-week in-situ study during which the participants (college students) carried out their normal day-to-day activities (going to class, studying, etc.). The experiment took place at a public university on the US west coast. We used a mixed study design so that each participant served as their own baseline to account for individual differences. The study consisted of a 1-week control session followed by a 3-week intervention session. We chose a period of 4 weeks so that the control and the intervention sessions spanned over the same days of the week, thus minimizing the influence of a given day’s schedule on the users’ daily activities and mood. To investigate how smiling, reflecting, and giving to others might impact users’ mood, we designed three experimental conditions:

- *Selfie:* participants would take a selfie daily while smiling;
- *Personal:* participants would take a photo daily of something that makes themselves happy;
- *Other:* participants would take a photo daily of something that they believe would make another person happy and then they would send it to that person.

### Materials

**Fig. 1:**
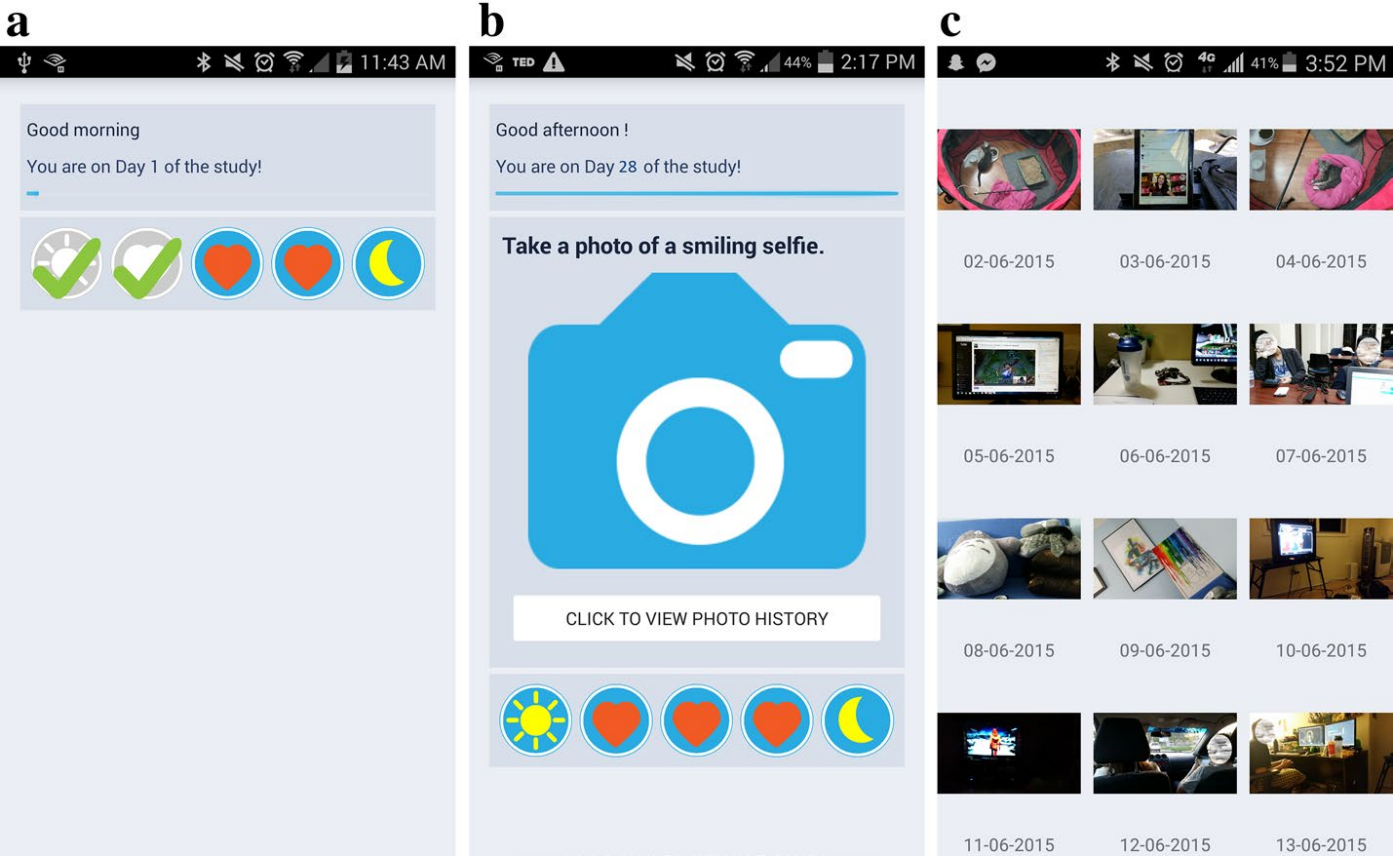
Screenshots of SurveyApp and MettaApp. **a** SurveyApp Homepage; **b** MettaApp homepage; **c** photo history on MettaApp

We developed two Android applications, *SurveyApp* and *MettaApp*, as experimental platforms for the control session and the intervention session respectively. The SurveyApp was designed to collect users’ mood in the control session. Figure 1a is the home screen of SurveyApp. The app includes five main tasks: a morning survey, three mood surveys during the day and an evening survey. Each task is visualized by a colored icon. If users have finished any of the tasks, the corresponding colored icon is greyed out and checked. Users could also see which day it is of the experiment.

The MettaApp was designed to collect users’ moods and enabled them to take photos in the intervention session. The MettaApp was built on top of the SurveyApp. It extended the functions of the SurveyApp and included an additional photo function (see Fig. 1b). A user could take one photo per day by clicking the camera button. The button is then replaced by the photo that the user has taken. Users could check all their photos by clicking the button “Click to view photo history” (Fig. 1b). Figure 1c shows the photo gallery in the timeline.

### Participants

We recruited 57 participants on campus by making announcements in classes and placing advertisements on Facebook. Ten participants withdrew from the study during the control session due to system incompatibility issues and six withdrew during the intervention session due to personal reasons. In the end, 41 participants completed the entire study, including 14 in the Selfie condition, 14 in the Personal condition and 13 in the Other condition. All participants were undergraduate or graduate students who used an Android phone as their primary phone. The participants, 13 males and 28 females, were between 18 and 36 years old. They were assigned randomly to one of the three conditions: Selfie, Personal and Other. Table 1 shows the distribution of participants’ gender and major by experimental conditions. We categorize their majors by STEM (science, technology, engineering and mathematics) and others. At the end of the study, participants were compensated with $25.

**Table 1:** Demographic information of participants

Condition	Gender		Major	
	Male	Female	STEM	Other
Selfie	5	9	5	9
Personal	4	10	6	8
Other	4	9	5	8
Total	13	28	16	25

### Procedure

Before the study, we invited participants to the laboratory for an informational meeting, to fill out a general survey and to sign informed consent. We assisted them with installing the SurveyApp at the beginning of the control session and the MettaApp at the beginning of the intervention session on their own Android phones from a given link. During the study, users reported their mood during each day, three times per day. In the evening survey, we also asked participants to indicate whether there was any significant event that happened to them that day at work or at home that affected their mood or stress level. If so, we asked them to briefly describe it. The above tasks were completed on the SurveyApp during the control session and on MettaApp during the intervention session. Starting from the beginning of the intervention session, participants used the MettaApp to take photos according to the condition to which they were assigned. At the end of the study, participants returned to our laboratory for an exit interview.

#### Mood Sampling

**Fig. 2:**
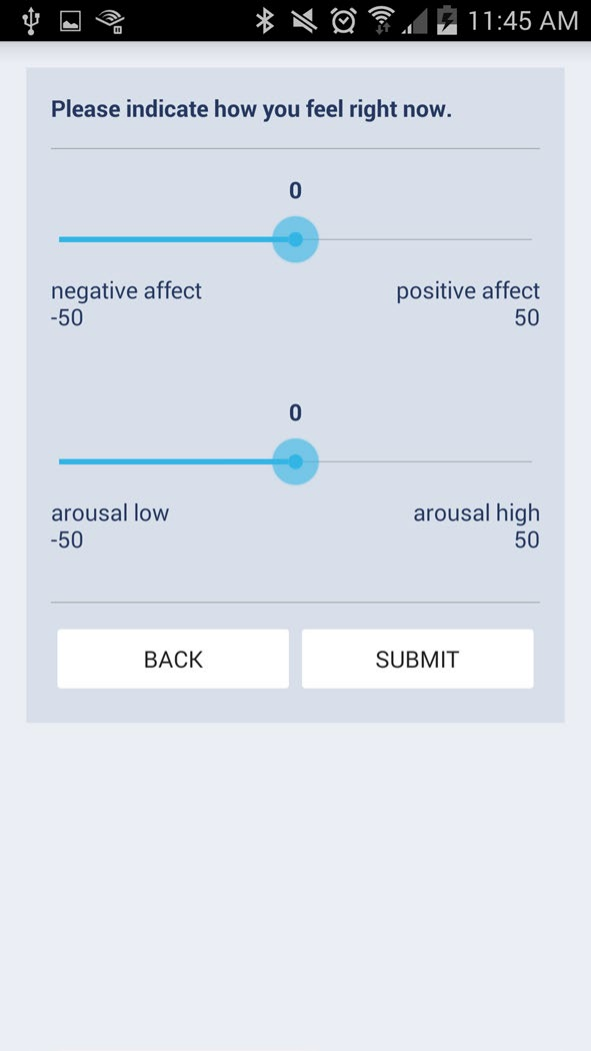
Mood sampling page

We obtained users’ moods using a visual representation of Russell’s Circumplex model (Russell 1980). This model measures users’ mood in two dimensions that are orthogonal: valence (i.e., how positive one feels) and arousal (i.e., how intense the feeling is). Even though the initial goal was to improve users’ positive affect, we assessed users’ mood in both valence and arousal, since we wanted to gain a more nuanced understanding of the effect that our interventions might have on users’ positive affect. We instructed participants on the meaning of the measures during the pre-study informational meeting. During the control session, the mood sampling requests were triggered via a notification on the SurveyApp three times during each day: in the morning (approximately 10 a.m.), in the afternoon (approximately 2 p.m.) and in the evening (approximately 7 p.m.). During the intervention session, the mood sampling requests were triggered on the MettaApp three times after a photo was taken: 5 min, 1 and 3 h after the photo. Figure 2 shows the interface from where participants input their mood using two sliding bars. On the upper sliding bar, participants indicated the valence of their feeling “right now” using a range of −50 (negative) to +50 (positive). On the lower sliding bar, they selected a value for their arousal between −50 (arousal low) and +50 (arousal high). We chose a large range between −50 and +50 to to maximize the opportunity to capture nuanced responses given the constraints of the limited screen space on smartphones. We logged the time when they answered the probes.

#### Taking Photos

Participants took one photo every day using MettaApp during the 3-week intervention session from Weeks 2 to 4. They took photos following the instructions they received: the Selfie group took photos while smiling, the Personal group took photos of things that made themselves happy and the Other group took photos of things that would make other people happy and then they sent the photos to others. For the Other group, they could choose their preferred methods to send photos, e.g., text message, email, or social media apps. Participants were shown the photo they took that day every time they opened the app and they were also able to view all the photos they had taken in the previous days. The photos were uploaded and backed up to a secure server and were only accessible to the participant and the research team.

#### Exit Interview

After the study, each participant returned to the laboratory for an individual exit interview. During the semi-structured interview, we asked participants their daily frequency of using the application, and their experiences while using the MettaApp. We also reviewed the photos with the participants and asked them to show the photos with which they felt most happy and to discuss the reasons. Then we encouraged them to contribute ideas about designing technology for emotion intervention. Finally, participants were instructed to uninstall the experimental applications from their smartphones. After uninstallation, the applications were deactivated and could no longer collect any data from participants. Each interview took about 25 min and we audio-recorded all the interviews.

## Findings

We collected the following types of data: (1) the valence and arousal obtained from daily mood sampling from the phone during the control and intervention sessions, (2) photos uploaded during the intervention session, and (3) interview data which were then transcribed. This section reports both quantitative and qualitative results. We present results of quantitative analyses on the intervention effects on mood and the comparison of intervention effects among the three conditions. We then present our findings of how the photo-taking made the participants happy by qualitatively analyzing their interview transcripts. Finally, we compared the participants’ photos in the three conditions through visual inspection and coding.

### Intervention Effects on Mood

We collected 2897 mood measures from experience sampling. The mean valence was 15.37 (SD = 19.8, Max = 50, Min = $-50$) and the mean arousal was $-2.76$ (SD=25.1, Max = 50, Min = $-50$). To examine the effects of the intervention of taking photos, we conducted a Linear Mixed-Effects Model (LMM) analysis in SPSS. LMM handles random and fixed effects and was used since our participants had repeated measures over days of their mood responses, from the mood sampling. We averaged the valence responses at 5-min, 1- and 3-h for each day and similarly, arousal responses at 5-min, 1- and 3-h for each day. We thus had 985 total responses. Our dependent variables were Valence (the daily averaged valence responses) and Arousal (the daily averaged valence responses), analyzed in separate models. With Condition (Selfie/Personal/Other) as a between-subjects variable and Intervention (before/after the intervention) as a within-subjects variable, we entered an interaction term of Condition *x* Intervention. These variables were entered as fixed effects. Participants were entered as random effects.

Table 2 shows the mean Valence and Arousal in the three conditions before and after the intervention. For Valence, we found a significant main effect of Intervention: F(1926) = 10.03, p = 0.002, Mean_Before = 13.64, SE = 1.64; Mean_After = 16.65, SE = 1.54. The main effect of Condition and the interaction between Intervention and Condition were not significant. Thus, participants in all three conditions rated their valence higher with the photo interventions.

For Arousal, we found a trend of significant Condition *x* Intervention interaction: F(2922) = 2.63, p = 0.072. Participants in the Other condition reported lower arousal after the photo intervention (see Table 2). The main effect of Intervention and the main effect of Condition were not significant. Based on Russell’s circumplex model of mood mapping (Russell 1980), we refer to the lower arousal scores as reflecting a calmer mood. Thus, taking photos is associated with participants in the Other condition becoming calmer compared with the Personal condition.

An LMM analysis of the temporal effects of rating Valence and Arousal 5 min, 1 and 3 h after the photo-taking showed no significant difference with time of response.

**Table 2:** Mean and SD of participants’ valence and arousal in the control session and the intervention session

Condition	Session	Valence		Arousal	
		Mean	SD	Mean	SD
Selfie	Control	12.16	13.48	−2.30	17.47
	Intervention	13.55	16.28	−3.60	21.09
Personal	Control	12.62	15.29	2.97	17.17
	Intervention	16.00	18.55	3.45	21.67
Other	Control	16.72	14.80	−5.74	22.40
	Intervention	19.84	15.6	−11.05	21.89

### Qualitative Results: How do Photos Make People Happy?

We then analyzed the transcripts of the exit interviews to understand why and how taking different types of photos influenced people’s mood. Three researchers transcribed the audio recordings of the interviews. We then used grounded theory (Strauss and Corbin 1994) to analyze the interview data. Table 3 summarizes the main themes derived from the interview data.

**Table 3:** Themes of coded qualitative data

Condition	Themes	Number
Selfie	Changed mood, due to feeling more confident, comfortable, or creative in smiles	5
	Constraints: brought more stress; inconvenient; repetitive smiles became boring	4
Personal	Became more mindful, reflective and appreciative	9
	Became aware that things around them served as important sources of happiness	5
Other	Receiving responses from the recipients of the photos made participants happy	7
	Helped the participants communicate their current situation	6
	Took photos of things that embedded shared memories	4
	Connecting with strong ties reduced their stress	6

### Selfie Condition

Five out of the fourteen participants in the Selfie condition observed changes in their smile and mood over the course of the 3-week photo intervention session. Some participants felt more confident over time about themselves taking selfies, such as P29: *“As days went on, I got more comfortable taking photos of myself. If you feel good about yourself, then [a] selfie would be a way to capture that.”* P46 reported that he became better at taking smiling selfies and noticed less stress on his face. P12 looked back on her selfies from time to time and became more creative in her photos by making gestures while taking the selfie. P40 reported that she sometimes reflected on the moment when she smiled. Two participants reported that even fake smiles lifted their mood up. As P29 said, *“It made me feel good, thinking, ‘this is probably how I look like for the rest of the day.’... It’s a way of telling me that I could get through the day no matter what happens. One of the photos was taken when I found out my friend passed away. That was a fake smile. I was depressed. I figured [that] if I can see myself smiling in the picture, things would be okay for the day.”*

Participants also reported some constraints in taking the photos. First, fake and forced smiles sometimes brought them stress (N = 4). A previous study (Nass et al. 2005) shows that the emotions induced during intervention should match users’ genuine affect e.g., happy/energetic, upset/subdued. This might explain some participants’ negative feedback of smiling selfies. Second, some participants found it inconvenient to find a private place to take a smiling selfie (N = 3). *“Sometimes it was difficult because I was not comfortable taking photos of myself in the public places. Not easy to fit into my day”*, said P21. Third, for participants who always took the selfie at the same place, repeatedly taking the same photo became boring (N = 2).

### Personal Condition

In the Personal condition, most participants became more mindful, reflective, and appreciative by taking photos. The most frequently reported reason (N = 9) for being happier after the 3-week photo session was that the photos helped them to be reflective. They thought more carefully about the source of their happiness. *“I do not use a social media app to reflect on something happen[ing] on a particular day. Using this app made me think of something [that] made me happy, reminding me of things that made me happy”*, said P27. A theme that emerged in the data was that participants started to realize happiness could come from things in their lives that they usually take for granted. For example, P31 commented, *“They just open my eyes and made me realize what makes me happy. Those are simple things that I never thought about before. Just like everyday objects and places in my room. They are places that made me content and stress-free at that time. Not big, but it does have an impact.”* For P51, he realized that he was happy because of social connections and experiences. *“All the photos had special meanings for me: hanging out with friends, socializing with people I care about, enjoying the experience, like coffee or a movie. I took one immediately after watching a movie with my roommate.”*

Some participants started to pay attention to their family members. As P28 said, *“Instead of going routinely and mechanically during the day, I stop and look around for something that makes me smile. I didn’t consciously do that before. I find that happiness is close to me. A lot them are my family and my pet. For my family, I didn’t think of them as a daily source of happiness. I usually took them for granted.”* Some became mindful of small things around them. For example, P25 started to consciously notice something that was nice even if it was in the background that she would not have noticed otherwise. She photographed mostly flowers that she walked by during the day and took two photos of her cat.

Realizing that things around them served as an important source of happiness, some participants reported that they became more appreciative (N = 5). As P36 said, *“They make me appreciate the small things in my life -things that I would normally not notice or take for granted. There are some photos of family members, reminding me of a reason to live for and making me happy. Sometimes I took pictures of my laptop. It helps me do well in school and brings a lot of convenience to my life. It made me happy. I don’t get excited, but feel grateful. It’s good that I have one.”* P23 and P24 reported that they started to cherish the time with their friends or significant others and felt grateful for their company.

### Other Condition

In the Other condition, 95 % of photos were sent to strong ties, i.e., family members, friends and significant others. Most participants reported that they thought more of and felt more connected with strong ties during the photo intervention period than before. As P20 mentioned, *“I don’t talk to my dad every day. But when I sent the photo to him, it made him happy, as a way of communication.”* P18 also reported reflecting and appreciating more of her life. *“I feel taking the photos made me realize lots of simple things not only made other people happy but also made myself happy.”*

Seven participants mentioned receiving a response from the recipients of the photos. The participants became more satisfied because they became aware that they made the person who received the photo happy. *“It was fun to send stuff to my girlfriend to make her laugh. Seeing her reactions will always make me smile,”* said P44. Similarly, P43, who often sent pictures to her boyfriend reported, *“I usually send photos of what I was doing or watching, or something that happened that day, for example, an advertisement or a flyer for a show. He always responded: ‘that’s really cute!’ ‘That’s awesome, can we see the show?’ That made me happy and showed how supportive he was and always had the same amount of excitement as I had.”* P16 sent a photo to her friend as a birthday gift. *“She has a crush on someone and I took the photo on her birthday. I messaged her this photo greeting her happy birthday, and she said that made her day. I was really happy.”*

Many participants reported that by taking and sending pictures of their present moment, they made their strong ties happy. The photos helped the participants communicate their current situation, e.g., how they were feeling, what they were working on and what environment they were in (N = 6). As P35 said, *“I was at the library and decided to show my mom how hard I was working. So, I took a picture of my notes and textbooks and then sent it to her. It made her happy knowing the effort I was putting in.”* P37 took most of the photos for her mother and sister, who were in a different country: *“For my mom, it’s mostly what I’m doing. Some pictures might look boring, but she was happy knowing what I was doing.”*

Participants also took photos of things that embedded shared memories (N = 4). For example, P43 intentionally took pictures to make his girlfriend happy; *“There was something we joked about before. It was the personal connection that gave the meaning. I was not taking pictures [that would be] super meaningful for others.”* P30, who usually took photos of her mother’s favorite things, said, *“It was nice to have something to send to somebody every day. I usually sent [them] to my mom. Sometimes she laughed at the pictures: ‘thanks for thinking of me today’... It let her know something reminded me of her and that I was missing her.”*

Participants also mentioned that connecting with strong ties reduced their stress. *“People can be comforted by these sort of photos. If someone is feeling depressed, the first thing they need is connection,”* described P15. This trend is more visible for participants who are international students and whose family is physically far from them (N = 4). *“Just the action of sending a photo already made my parents happy, because they feel more assured about my studies and my life, or because I’m thinking about them. When I felt stressed with my studies, the intimacy from interacting dispelled the loneliness, making me appreciative and relieved. That takes me away from the stress,”* explained P10. Connecting with strong ties may be one explanation of why participants in the Other condition reported feeling much less aroused after taking and sending photos.

### Analyzing the Photos

We collected a total of 692 photos from participants, comprised of 271 in the Selfie condition, 227 in the Personal condition and 194 in the Other condition. Two researchers independently coded the photos in the three conditions. For the Selfie condition, they coded the locations where the selfies were taken, e.g., home and school. For the Personal and the Other conditions, they coded the content of the photos, e.g., friends and food. With an agreement rate of 96.1 % on the coded labels initially, the two researchers then reached a consensus on the rest of the photos mediated by a third researcher.

Table 4 summarizes the locations and their distribution. For the selfies, most of them were taken at home (65.3 %). The rest were taken in cars (10.0 %), at study areas (3.1 %), or at restaurants (2.1 %). Why were most smiling selfies taken at home? The interview data reveal that many participants started a day by making a smiling selfie at home (N = 4) or signaled the end of a day with a selfie when arriving at home (N = 3). Others mentioned that they tried to take smiling selfies in private places rather than public places such as in the classroom or at workplaces in order not to be embarrassed. As P14 said, *“I always make sure no one is around, and I look presentable.”* Always taking smiling selfies with the same facial expression at the same place could explain why some participants felt bored by taking the selfies. By contrast, some participants preferred to take selfies with a background that embedded particular meanings, such as at a banquet, before a wedding and after a satisfying haircut. This suggests that encouraging users to smile during meaningful events and at a variety of occasions can help reduce the perception of boredom in the smiling selfie exercise.

**Table 4:** Distribution of the locations where the smiling selfies were taken

Location	Number	Percent of total
At home	190	65.3
In car	29	10.0
Outdoors	22	7.6
At work/study place	9	3.1
In restaurant	6	2.1
Miscellaneous	15	5.1
Total	271	100

For the Personal and the Other conditions, we coded the content of the photos to investigate what kinds of things participants indicated as making themselves or other people happy. Table 5 lists the themes of the two conditions ranked by proportion. Food ranks top among all photo themes, 19.4 % in the Personal condition and 22.2 % in the Other condition. It seems that food made participants themselves happy, as well as their strong ties. A social theme, which includes family, friends and significant others, is another common theme in both the Personal condition (17.2 %) and the Other condition (8.2 %). Photos of personal theme were frequently taken by participants, 16.3 % in the Personal condition and 20.6 % in the Other condition. The personal theme includes personal spaces where people live, study and work, as well as personal items such as toys, pictures, figurines and ornaments. Photos of this theme were frequently sent by participants in the Other condition to their strong ties. They were used as a communication channel to inform the receiver about their everyday life and thus increase mutual awareness and intimacy between the sender and the receiver. By contrast, participants in the Personal condition took photos of places where they live, study and work, which could serve to remind them that happiness exists in their surroundings—the simple and tiny things around them. For the entertainment photos, such as video games, Youtube videos and Netflix, participants in the Personal condition took more than the Other condition (15.0 vs. 9.8 %). For nature themes, such as flowers, the sea and tress, the Other condition has a slightly larger share than the Personal condition (18.0 vs. 14.1 %). Some selfies were taken by participants in the Other condition and these were mainly sent to significant others (5.2 %).

**Table 5:** Distribution of photo themes in the personal and other conditions

Personal condition			Other condition		
Theme	Number	Percent of total	Theme	Number	Percent of total
Food	44	19.4	Food	43	22.2
Social	39	17.2	Personal	40	20.6
Personal	37	16.3	Nature	35	18.0
Entertainment	34	15.0	Entertainment	19	9.8
Nature	32	14.1	Social	16	8.2
Pet	20	8.8	Technology	11	5.7
Technology	10	4.4	Selfie	10	5.2
Urban	5	2.2	Beauty	6	3.1
Art	3	1.3	Art	5	2.6
Beauty	2	0.9	Spiritual	4	2.1
Spiritual	1	0.4	Urban	3	1.5
Selfie	0	0.0	Pet	2	1.0
Total	227	100.0	Total	194	100.0

## Discussion

The results suggest that *any* photo-taking with the intent to increase one’s happiness can increase positive affect, specifically photos intended to promote happiness via smiling self-expression (selfies), those taken of things to make ones’ self happy, or those intended to make others happy. Moreover, sending photos to others makes people less aroused. As described earlier, based on Russell’s circumplex model of mood mapping (Russell 1980), we refer to the lower arousal scores in the Other condition as participants becoming calmer. Humans are social creatures. Connecting with strong ties helps people become calmer, especially for those who tend to cope with stress through emotional support (Cohen and McKay 1984). In fact, most of the photos taken were of things that connect the sender and the receiver, for example, those that document the current state of their life or embed shared memories. Seemingly small things can increase the intimacy of strong ties in online communication (Bales et al. 2011). On the other hand, taking photos that make people close to them happy further requires users to think beyond themselves to benefit others. As the research of (Seppala and Tomasello 2013) shows, depression and anxiety are linked to self-focus. When people make an effort to increase the happiness of other people, they are broadening their perspective beyond themselves. Other-focused attention and thinking about others has been shown to trigger a decrease in heart rate and skin conductance (Calvo and Peters 2014; Goetz et al. 2010).

We also asked participants in the interviews to compare MettaApp with photography apps on social media. Most participants (N = 22) mentioned the photos with MettaApp were mainly for themselves. They felt more comfortable expressing themselves in the pictures without being disturbed by external factors, such as impression management, or how others will perceive them. By contrast, photos posted on Instagram, Facebook, and Snapchat are mainly targeted for their social circle, which is sometimes hundreds of people. Participants would keep their audience in mind, take into account the likes and comments on social media and try to make the photos presentable and look perfect. For P36 in the Personal condition, taking a moment to stay centered in his life without social influence helped him rediscover the source of happiness in his life.

Further, we encouraged participants in the interviews to suggest future technologies that could enhance their happiness using photography based on their experience in this study. One recommendation that surfaced often from participants is to design technologies to help people review photos of happy moments in the past (N = 14). Such a technology could display the photos of happy moments to people when are experiencing a bad mood. Participants also imagined tools that could help them review happy moments at the end of the day for a better sleep, or at the beginning of the next day to start a day with positive energy. Reflecting emotions, especially positive emotions, is shown to help improve users’ mental well-being (Jaques et al. 2015; McDuff et al. 2012). Some participants suggested technologies that could pop up “happiness” photos at random times of the day to give them a surprise of positive reminiscence. Participants also suggested “smart” photography that detects mood automatically. With pervasive sensors and wearable devices that track users’ mood, future technology may send users “happiness” photos when it detects their negative mood (P27). Meanwhile, such technology could also recommend that a user take a photo to record the moment if the sensors have detected an increase in a user’s positive affect (P31).

## Limitations

This study focuses on three exercises instead of covering an exhaustive list from positive psychology. It may be worth exploring interventions that combine these conditions, such as taking selfies with strong ties in the photo or sending selfies to strong ties. It is also possible that the period of the photo intervention coincided with a period where our participants were more positive, or changes over time could have played a role in the results. However, the intervention occurred towards the end of the academic quarter when students generally experience more stress. So the fact that valence increased and arousal decreased for some people is contrary to what we would expect without any intervention, given the time when the study was conducted. Moreover, since people were tested over a period of time, experiencing different environments, the environment should play less of a role in influencing the results. In this study we did a within-subjects design, where each participant served as their own control. In future studies, to rule out changes over time that could affect the results, we could include a control group to further validate the findings of this study.

## Conclusions

We aimed to leverage the prevalence of smartphone photography along with theories of positive psychology to help college students become happier and reduce stress. To this end, we conducted a 4-week study with 41 participants to investigate the effects of taking *daily* photos using their smartphones in three conditions: the Selfie condition in which participants took a smiling selfie, the Personal condition in which participants took a photo of something that made themselves happy and the Other condition in which participants took and sent a photo of something to make another person happy. Quantitative and qualitative results show that participants in all three conditions became more positive after taking their assigned type of photo daily for 3 weeks. Some participants in the Selfie condition observed a more natural smile over time; participants in the Personal condition became more reflective and some participants reported that the photos led them to be more appreciative of the little things in their lives that made them happy. Participants in the Other condition became much less aroused (i.e., calmer) with photo-taking and some reported the increased intimacy and connection with strong ties as an important factor that can reduce anxiety, serve to pacify themselves and lead them to become more positive. Compared to photos posted on social media, participants felt more comfortable, conscious, and reflective when taking the photos. They also suggested future technology that could help them take and review photos of happy moments using mood-tracking sensors.

This paper provides empirical support on the feasibility of increasing users’ happiness by applying positive psychology to smartphone photography. It also contributes to the emerging field of positive computing by presenting reasons for how conducting exercises to promote happiness using mobile technology could help people enhance their mood. The findings can offer insights for designers to create systems that enhance emotional well-being.
